# Differential effects of social isolation on oligodendrocyte development in different brain regions: insights from a canine model

**DOI:** 10.3389/fncel.2023.1201295

**Published:** 2023-07-18

**Authors:** Huilin Hong, Chao Guo, Xueru Liu, Liguang Yang, Wei Ren, Hui Zhao, Yuan Li, Zhongyin Zhou, Sin Man Lam, Jidong Mi, Zhentao Zuo, Cirong Liu, Guo-Dong Wang, Yan Zhuo, Ya-Ping Zhang, Yixue Li, Guanghou Shui, Yong Q. Zhang, Ying Xiong

**Affiliations:** ^1^State Key Laboratory for Molecular and Developmental Biology, Institute of Genetics and Developmental Biology, Chinese Academy of Sciences, Beijing, China; ^2^Division of Life Sciences and Medicine, School of Life Sciences, University of Science and Technology of China, Hefei, China; ^3^State Key Laboratory of Genetic Resources and Evolution, Kunming Institute of Zoology, Chinese Academy of Sciences, Kunming, China; ^4^State Key Laboratory of Brain and Cognitive Science, Institute of Biophysics, Chinese Academy of Sciences, Beijing, China; ^5^College of Life Sciences, University of Chinese Academy of Sciences, Beijing, China; ^6^Bio-Med Big Data Center, Key Laboratory of Computational Biology, CAS-MPG Partner Institute for Computational Biology, Shanghai Institute of Nutrition and Health, Shanghai Institutes for Biological Sciences, Chinese Academy of Sciences, Shanghai, China; ^7^Beijing Sinogene Biotechnology Co., Ltd., Beijing, China; ^8^Institute of Neuroscience, Center for Excellence in Brain Science and Intelligence Technology, Chinese Academy of Sciences, Shanghai, China; ^9^Shanghai Center for Brain Science and Brain-Inspired Intelligence Technology, Shanghai, China

**Keywords:** social isolation, dog, oligodendrocyte, myelin, parietal cortex, blood-brain barrier

## Abstract

Social isolation (SI) exerts diverse adverse effects on brain structure and function in humans. To gain an insight into the mechanisms underlying these effects, we conducted a systematic analysis of multiple brain regions from socially isolated and group-housed dogs, whose brain and behavior are similar to humans. Our transcriptomic analysis revealed reduced expression of myelin-related genes specifically in the white matter of prefrontal cortex (PFC) after SI during the juvenile stage. Despite these gene expression changes, myelin fiber organization in PFC remained unchanged. Surprisingly, we observed more mature oligodendrocytes and thicker myelin bundles in the somatosensory parietal cortex in socially isolated dogs, which may be linked to an increased expression of ADORA2A, a gene known to promote oligodendrocyte maturation. Additionally, we found a reduced expression of blood-brain barrier (BBB) structural components Aquaporin-4, Occludin, and Claudin1 in both PFC and parietal cortices, indicating BBB disruption after SI. In agreement with BBB disruption, myelin-related sphingolipids were increased in cerebrospinal fluid in the socially isolated group. These unexpected findings show that SI induces distinct alterations in oligodendrocyte development and shared disruption in BBB integrity in different cortices, demonstrating the value of dogs as a complementary animal model to uncover molecular mechanisms underlying SI-induced brain dysfunction.

## Introduction

Social interactions are basic human needs, analogous to other basic needs such as nutrition or sleep (Baumeister and Leary, 1995; Cacioppo et al., 2011; Tomova et al., 2020), which offer safety and security, support offspring survival, reduce the need for energy expenditure, and provide a form of social reward (Eisenberger, 2012). Social interaction is important for people at all ages; a lack of or reduced social interaction, such as mandated measures during the COVID-19 pandemic, leads to socioemotional and cognitive deficits in early life (<5 years old) and an increased occurrence of psychiatric disorders such as depression and anxiety during adolescence and adult stages (Rutter et al., 2007; Cacioppo et al., 2011; Kennedy et al., 2016; Sonuga-Barke et al., 2017; Loades et al., 2020; Deoni et al., 2021; Pancani et al., 2021; Taheri Zadeh et al., 2021; Shuffrey et al., 2022). Magnetic resonance imaging and diffusion tensor imaging neuroimaging analysis revealed decreased white matter integrity in specific brain regions, including the prefrontal cortex (PFC), in children who experienced early social deprivation (Eluvathingal et al., 2006; Govindan et al., 2010; Bick et al., 2015).

The mechanisms underlying SI-induced brain structural and behavioral changes have been mostly studied in PFC using rodent models (Liu et al., 2012; Makinodan et al., 2012; Yamamuro et al., 2018; Xiong et al., 2023). For example, SI leads to reduced expression of the ErbB3 ligand neuregulin-1. The NRG1–ErbB signaling pathway, which is important for OL maturation, is linked to reduced medial PFC myelination in response to SI in juvenile mice (Makinodan et al., 2012). Since the developmental progression of various processes including myelination of different brain regions is distinct (Hong et al., 2022), different brain regions may react differently to SI. Uncovering those SI-induced molecular changes in different brain regions are critical to understand SI-induced brain dysfunction. However, it remains unclear how SI affects different brain regions at the molecular and cellular levels.

Domestic dogs (*Canis familiaris*), with a gyrencephalic brain structure similar to that of humans, have evolved complex and efficient cross-species emotional and social processing abilities during long history of coevolution with humans (Muller et al., 2015). In addition, our recent work has revealed conserved inter-regional protein expression patterns, especially myelination-related proteins, in the brain between dog and human (Hong et al., 2022). The domestic dog has been used for decades as experimental models in studies of neuroscience, cognition, evolutionary genetics, and diseases such as neurological and psychiatric disorders (Adams et al., 2000; Wang et al., 2013; Bunford et al., 2017; Liu et al., 2018; Cao et al., 2021). In particular, dogs are considered effective models for studying social behaviors and mental disorders caused by adverse early life experiences (Berns and Cook, 2016; Ogata, 2016; Bunford et al., 2017; Dietz et al., 2018).

To investigate the mechanisms underlying SI-induced brain dysfunction, we performed transcriptomic and immunochemical analyses of various brain regions from socially isolated and group-housed dogs during the juvenile stage. We found that SI decreased myelin-related gene expression specifically in PFC white matter, but myelin fiber organization remained unchanged. However, SI increased thickness of myelin bundles containing more fibers in the somatosensory parietal cortex (Par), possibly due to more mature oligodendrocytes. SI also disrupted blood-brain barrier (BBB) integrity in both PFC and Par, as evidenced by reduced expression of BBB component proteins. Consistently, myelin-related lipids were significantly increased in cerebrospinal fluid (CSF). These findings shed new light on the molecular and cellular mechanisms underlying the detrimental effects of SI on the brain.

## Materials and methods

### Dogs and housing conditions

Purebred healthy male Beagles were obtained from Beijing Marshall Biotechnology Co., Six male Beagle dogs (weight 2.9 ± 0.3 kg) from three different litters were maintained in a natural 12-h light-dark cycle (2 dogs in each cage). After weaning at postnatal day 51 (P51), 6 littermates were reared together in a cage until P60. Then, three males, one per litter, were housed together in one cage. The other three were housed individually in a quiet location within a building with minimum human activity for 4-weeks.

### MRI data acquisition and analysis

Five beagles (3 socially isolated and 2 group-housed littermates of the socially isolated dogs; one of the 3 group-housed dogs had a chip implanted under its skin, which made it unsuitable for MRI analysis; age 96.6 ± 0.49 days, weight 4.8 ± 1.59 kg) were scanned at 3T MRI scanner (MAGNETOM Prisma, Siemens Healthcare, Erlangen, Germany) with a home-made 4-channel Tx/Rx RF coil to obtain high quality structural MRI (sMRI) and diffusion MRI (dMRI). For sMRI, T2-weighted images were acquired at the same position and spatial resolution as T1-weighted images using the SPACE sequence, which utilized different flip angles to optimize contrasts for T2 sampling. The main scan parameters: FOV = 128 × 128 mm2; TE = 3.68 ms; TR = 2,370 ms; TI = 1,030 ms; FA = 8°; acquisition data matrix size 256 × 256. DWI was acquired using an EPI sequence with multiband acceleration. The main scan parameters were: voxel 1.2 mm isotropic, FOV = 120 × 120 mm^2^; TE = 86 ms; TR = 7,000 ms; FA = 90°; acquisition data matrix size 80 × 80, 64 diffusion gradients in different directions, and four b-values of 0 s/mm^2^, 1,000 s/mm^2^, 2,000 s/mm^2^, and 3,000 s/mm^2^, respectively. Each scan lasted about 30–45 min. For diffusion datasets, the original diffusion images were preprocessed using FSL, including motion and eddy-current corrected. FA, MD, RD, and AD maps were obtained using “dtifit” algorithms. The FA, AD, and RD diffusion maps of PFC and Par regions in cortical gray matter and white matter were compared between the SI and Ctrl group using a two-sample unpaired *t*-test.

### Brain dissection

Brains were weighted and placed ventral side up onto a chilled glass plate on ice. Upon receipt of the dog brain, the fresh tissue was immediately embedded in a gelatin matrix using a self-made mold (patent No. ZL 2022 2 0238374.7). The brain was positioned for coronal sectioning. In order to check for technical artifacts, 3D printing brain models were used as prefabrication. The very first rostral section was obtained from the olfactory bulb. The stereotaxic reference grid was 2 mm intervals. Sections were divided into left and right hemispheres by cutting along the midline using a long scalpel. The left brain was used for lipidomics, RNAseq, and biochemical analysis. All specimens and residual brains were stored at −80°C after frozen in liquid nitrogen. The right brain was used for staining after the sections were fixed in 4% PFA in PBS for at least 72 h. For detailed descriptions of different brain regions, we referred to the supplement of the book named The Beagle Brain in Stereotaxic Coordinates (Palazzi, 2011). To ensure consistency, all dissections were performed by Dr. Huilin Hong and Dr. Hui Zhao.

### Immunohistochemistry of dog brain tissue and imaging

The animal was anesthetized with xylazine/ketamine or isoflurane and perfused with 4% PFA in PBS. The right brain hemisphere was removed and fixed in 4% PFA in PBS overnight. For cryosections, samples were transferred to 30% sucrose solution, embedded in optimal cutting temperature compound (OCT) for at least 2 days and stored at −80°C. Coronal dog brain sections (10 μm) were cut by cryostat (Leica). Sections were stored free-floating in cryoprotective solution (25% ethylene glycol, 20% glycerol, in PBS). High resolution and contrast myelin staining was achieved using the gold phosphate derivative, TrueGold Kit (BK-AC001, Oasis Biofarm Inc., Hangzhou, China), following published protocols (Schmued et al., 2008). For most staining, sections are permeabilized and blocked for 1 h with 0.2% triton-x-100, 10% Fetal Bovine Serum (FBS), and 5% Bovine Serum Albumin in PBS, and then incubated overnight at 4°C with the primary antibodies. Primary antibodies were diluted in 0.2% PBST and applied overnight at 4°C. The primary antibodies we used included rabbit anti-MBP (ab7349, 1:1,000), mouse anti-CC1 (OP80, 1:500), mouse anti-NeuN (ab104224, 1:500), rabbit anti-MYRF (OB-PRB007-02, 1:300), and guinea pig anti-Sox10 (OB-PGB001, 1:300), guinea pig anti-AQP4 (OB-PGP0016, 1:500), Goat anti-GFAP (ab53554, 1:1000), rabbit anti-laminin (L9393, 1:200), rabbit anti-occludin (71-1500, 1:500), rabbit anti-claudin1 (ab15098, 1:500). The sections were then washed with 0.2% PBST, and subsequently incubated with Alexa Fluor tagged secondary antibodies (1:1,000) for 2 h at RT.

Conventional confocal images of MBP bundles were collected at 488 nm with a Leica TCS SP8 confocal microscope using a 40× oil objective. Confocal stacks (z-step size = 1 μm) were processed with ImageJ software (National Institutes of Health). For MBP staining images, whole slide scans of tissue were collected with PerkinElmer Vectra Polaris using a 20× or 40× objective; 3D rendering and visualization were processed with Imaris 6.5 software.^1^

### Quantitative analysis of oligodendrocytes and myelin bundles

Oligodendrocyte density and morphology in dog brain were quantified as previously described (Makinodan et al., 2012; Tanti et al., 2018). Ratios of OL subgroups and morphological changes of OLs in white matter of the PFC and Par were quantified from 3 animals in each group. To quantify the number of the cellular protrusions of CC1^+^ OLs, over 90 CC1^+^ OLs from 3 animals of each group were characterized (the number of quantified CC1^+^ OLs in each animal of group: Controls, 41/33/30; SI, 41/39/39). The number of OL protrusions between groups was analyzed using one-way ANOVA and the Bonferroni test. The number of CC1^+^, Sox10^+^ OLs was quantified in a rectangle area of 461 × 263 μm using ImageJ.

To quantify the fluorescence intensities of MBP labeled myelin bundles composed of myelin fibers, three regions of interest of 0.06 mm^2^ were selected in layers 2/3 of the gray matter per sample for analysis by ImageJ. Statistical significance was calculated with two-tailed Student’s *t*-test. Data are presented as means ± SEM.

### RNA extraction

Tissue slices were taken from the medial PFC, Amygdala, Hippocampus, parietal lobe, occipital lobe, and flash frozen for subsequent processing. A bead mill homogenizer (Bullet Blender, Gingko Biotech) and chilled stainless-steel beads (SSB14B, Next Advance) were used to lyse the pulverized brain tissue. Total RNA was extracted using a non-phenolic procedure (RNeasy Plus Mini Kit, Qiagen), followed by DNase treatment (TURBO DNase, Ambion) as per product instructions. RNA was reverse transcribed with SuperScript™ III first-strand synthesis system for RT-PCR (Invitrogen, 18080-051) and quantitative real-time PCR (qPCR) was performed using KAPA SYBR^®^ FAST qPCR (KAPA, KK4601) at Stratagene Mx3000P Agilent technologies.

### Transcriptomic analysis

After the library was constructed, we used Qubit 2.0 for preliminary quantification, diluted the library, and then used Agilent 2100 to detect the size of the insert in the library. RNA-seq was performed by Novogene using an Illumina NovaSeq 6000 platform by PE150 sequencing strategy. After using fastp to trim reads to obtain high-quality reads, we generated at least 12 G of clean data. The paired-end reads were mapped to the Canis lupus familiaris reference genome (ROS_Cfam_1.0) using Hisat2. The sort command in Samtools was used to convert sam files to bam files. For improved gene-level analysis, StringTie was used to assemble and quantify the transcripts in each sample using the annotation gtf (ROS_Cfam_1.0.105) file for the Canis lupus familiaris reference genome. Then, the R package IsoformSwitchAnalyzeR was used to assign gene names to transcripts assembled by StringTie and estimate reads counts of gene level summaries, which can be particularly helpful in cases where StringTie could not perform assignment unambiguously. To ensure high confidence results, only the genes annotated as “protein coding” in Biomark and supported by more than 2 samples were used for subsequent differential expression analysis. We used DESeq2 to normalize the count matrix and analyze differential expression (fold change ≥1.5 and adj *p* ≤ 0.05).

### qPCR

Extracted RNA was reverse transcribed using an iScript™ cDNA Synthesis kit (BIO-RAD), followed by qPCR using KAPA SYBR(R) FAST kit (Roche, KK4601) on a Real-Time QPCR System (Agilent). The relative mRNA expression levels were analyzed according to the ΔΔ Ct method (Livak and Schmittgen, 2001). *GAPDH* was used as the reference gene. The genes and primers used for qPCR are listed in Supplementary Table 2. For validation of RNA-seq results, we compared the qPCR results of representative genes including *Aqp4*, *Uqcrh*, *Ndufa12*, *Cox7a2l*, *Erbb3*, *Egfr*, *Mobp*, *Ecsit*, *Ptp4a2*, *Adora2a*, *Arnt*, *Myo5a*, *Dusp19*, *Lrrc3b*, *B3galt6*, and *Cntn2* with RNA-seq data using Pearson correlation test.

### Functional enrichment analysis

Functional enrichment analysis was performed using the R package ClusterProfiler (4.4.1) (Wu et al., 2021) with default parameters (*p*-value cutoff = 0.05, *q*-value cutoff = 0.2). To ensure the accuracy of the enrichment test, only the genes involved in the differential expression analysis in the previous step were used as background. We examined all Gene Ontology terms from the latest version of “org.Cf.eg.db” and KEGG pathways from the official API.

### GSEA

Target gene sets were derived from experimentally validated mouse oligodendrocyte lineage markers (Marques et al., 2016). We assessed enrichment of these markers in transcriptome data using permutation testing with 100,000 iterations. The visualization of enrichment results relied on the plotting functions that come with ClusterProfiler (4.4.1) (Wu et al., 2021), except that the specific KEGG pathway maps relied on the Pathview web tool.^2^

### Lipidomics analysis

Lipids were extracted according to a modified version of the Bligh and Dyer’s protocol (Lam et al., 2021). The CSF lipidome was quantified using a high-coverage targeted lipidomic approach as described previously (Lam et al., 2021). All lipidomic analyses were conducted on a system comprising an Exion-UPLC coupled with a 6500 Plus QTRAP that runs Analyst v.1.6.3 (Sciex). All quantifications were conducted using internal standard calibration. Levels of short-, medium-, and long-chain TAGs and DAGs were calculated by referencing to spiked internal standards of TAG(14:0)3-d5, TAG(16:0)3-d5, TAG(18:0)3-d5, d5-DAG17:0/17:0, and d5-DAG18:1/18:1 from Avanti Polar Lipids. Free cholesterols and cholesteryl esters were analyzed as described previously with d6-cholesterol and d6-CE18:0 cholesteryl ester (CE) (CDN isotopes) as internal standards.

### Statistical analyses

Statistical significance between groups was determined by two-tailed Student’s *t*-test, whereas multiple comparison between genotypes was determined by one-way ANOVA with a Tukey *post-hoc* test and two-way ANOVA. Asterisks above a column indicate comparisons between a specific genotype and control, whereas asterisks above a horizontal line denote comparisons between two specific genotypes. ns denotes *p* > 0.05; *indicates *p* < 0.05; ^**^denotes *p* < 0.01; ^***^indicates *p* < 0.001.

## Results

### SI induces reduced expression of myelin-related genes in PFC white matter

To elucidate the effect of SI on brain development, we designed an experiment of SI of beagle dogs from 2 month after weaning to 3-month old. Specifically, three two-month-old beagle dogs (one from one litter) were socially isolated for 1 month, while the other three corresponding littermates were raised together in the same cage (Supplementary Figure 1). To examine which brain regions were affected by SI, we performed transcriptomic analysis of the prefrontal cortex (PFC) and parietal cortex, as well as the subcortical hippocampus and amygdala (Supplementary Table 1). The gray matter and the white matter of different cortices were analyzed separately.

**FIGURE 1:**
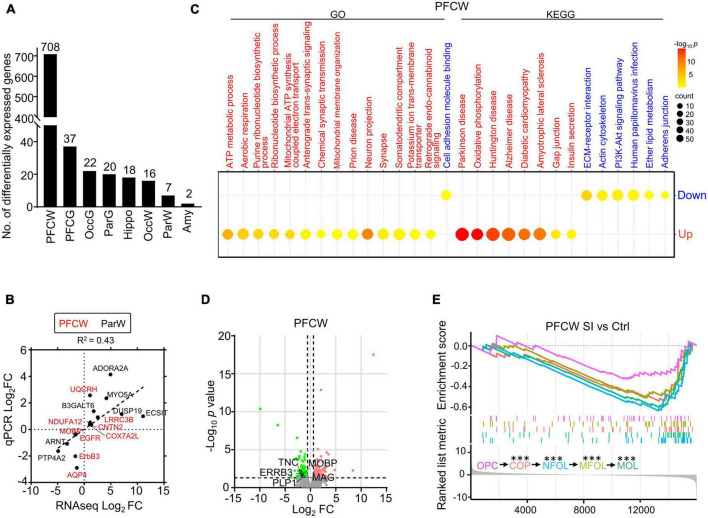
Social isolation (SI) leads to decreased levels of myelin-related gene expression and lipids in the white matter of the prefrontal cortex. **(A)** The number of differentially expressed (DE) genes in each brain region of SI dogs compared with group-housed controls. **(B)** The dot plot shows RNAseq and quantitative real-time-PCR (qRT-PCR) values of select genes with significant changes in different regions. **(C)** Bubble heatmap shows enriched GO and KEGG pathways of SI induced up- and down-regulated genes in the white matter of PFC (Fisher’s exact test, fc > 1.5, adjusted *p* < 0.05). Size and color of bubbles indicate fold enrichment for that pathway and adjusted *p*-value, respectively. Upregulated pathways are in red, downregulated pathways are in blue. **(D)** Volcano plots of differentially expressed genes in white matter of PFC after SI. The cutoff values were set at fc > 1.5 or fc < 0.67 and *p* < 0.05. Significantly down-regulated myelin-related genes are highlighted in green. **(E)** GSEA plot shows enrichment of different stage-specific markers, most of which are significantly down-regulated in PFC of socially isolated dogs compared with control. ***p < 0.001.

We found that the white matter of the PFC showed much more differentially expressed (DE) genes than the other regions after SI. Specifically, we detected 708 and 37 DE genes in the white matter and gray matter of the PFC, respectively, compared with 7 and 20 DE genes in the white matter and gray matter of the parietal cortex (Par) (Figure 1A). The representative DE genes from the PFC and Par, including *myelin-associated oligodendrocyte basic protein* (*MOBP*), *aquaporin 4* (*AQP4*), and *adenosine a2a receptor* (*ADORA2A*), were independently verified by RT-PCR (Figure 1B; Supplementary Tables 2, 3), validating the quality of the transcriptomic data. Gene Ontology (GO)/Kyoto Encyclopedia of Genes and Genomes (KEGG) analyses showed that the significantly increased genes in the PFC white matter of SI dogs are involved in ATP metabolic processes and neuron projections, while the decreased genes are involved in the extracellular matrix (ECM)-receptor interaction and cell adhesion (Figure 1C). No GO/KEGG pathways were identified in other regions due to a limited number of DE genes.

Although the myelin-related pathway was not enriched by GO/KEGG analysis, several myelin-related genes, including *MOBP* and *proteolipid protein 1* (*PLP1*), were significantly downregulated in the PFC white matter of SI dogs compared with control dogs (Figure 1D). To determine which stage of the myelination process was affected by SI in PFC, we performed gene set enrichment analysis (GSEA) of RNAseq data, which assessed the distribution of predefined gene sets. We utilized 50 previously published marker genes for each of the five cell types representing different stages of the myelination process (Marques et al., 2016). All markers for oligodendrocytes (OLs) at later maturation stages including differentiation-committed oligodendrocyte precursors (COPs), newly formed oligodendrocytes (NFOLs), myelin-forming oligodendrocytes (MFOLs), and mature oligodendrocytes (MOLs) were downregulated (Figure 1E). Together, our findings demonstrated that SI leads to reduced OL maturation in the white matter of PFC.

### SI induces thicker myelin bundles with more fibers in the Par but not in PFC

**FIGURE 2:**
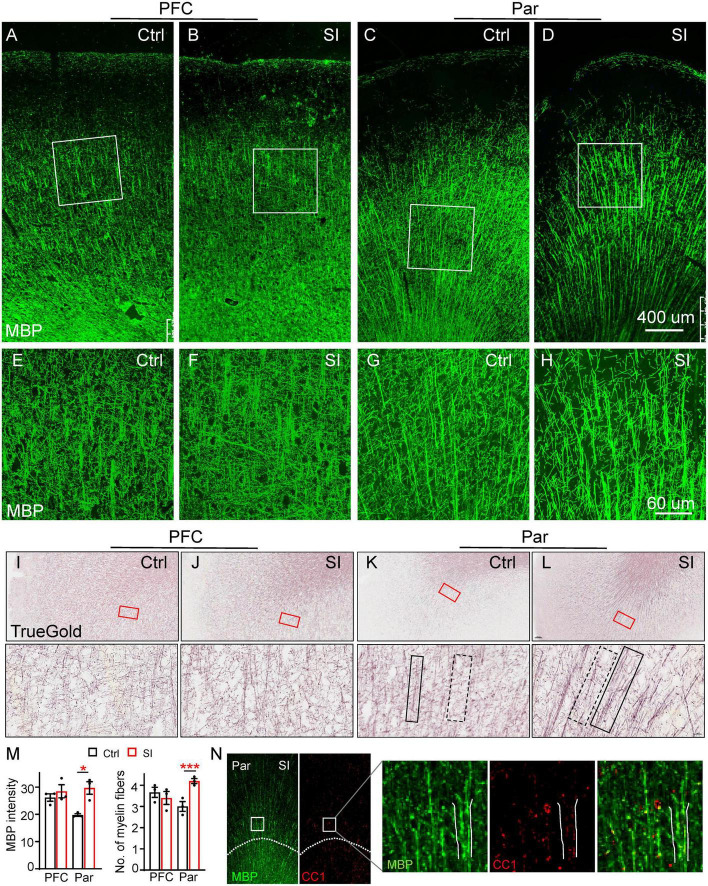
Social isolation (SI) of juvenile dogs results in the disorganization of myelinated fibers in the gray matter of the parietal cortex. **(A–D)** Confocal images of myelinated fibers labeled by MBP (green) in the PFC and Par of socially isolated and group-housed dogs. Scale bar, 400 mm. **(E–H)** Three-dimensional reconstruction images of MBP staining of Par and PFC regions boxed in the corresponding panels **(A–D)**. Scale bar, 60 μm. **(I–L)** Images of myelinated fibers labeled by TrueGold kit in PFC and Par of socially isolated and group-housed dogs. Scale bar, 10 um. No obvious changes of myelinated fibers in the gray matter of PFC but disorganized myelin fibers in Par gray matter were observed after SI. **(M)** Statistics of MBP intensity in panels **(A–D)** and the number of myelin fibers for each bundle in panels **(E–H)**. *p < 0.05; ***p < 0.001. **(N)** Co-staining images of MBP (green) and CC1 (red) in Par of socially isolated and group-housed dogs.

To examine the effects of SI-induced reduction of myelin-related gene expression on myelin, we labeled myelin fibers with an MBP antibody and the TrueGold dye, a gold phosphate derivative (Schmued, 1990). The organization of MBP-labeled myelin fibers in the gray matter of PFC and Par was different in that there was an obvious radial organization of myelin fibers from the white matter to the gray matter of the Par but not the PFC (Figures 2A–D). No obvious changes in myelin fibers in the PFC were observed after SI. However, the MBP-labeled myelin bundles by wide field microscopy were markedly thicker with parallel fibers in the Par after SI (Figures 2C–D). High magnification images showed thicker myelin bundles consisting of more parallel fibers and fewer myelin fiber crosses between longitudinal myelin bundles in the Par after SI (Figures 2G, H), but no changes in the PFC (Figures 2E, F). Specifically, the MBP intensity of myelin bundles and the number of myelin fibers in myelin bundles were significantly increased (Figure 2M). Similar to MBP staining, TrueGold staining showed thicker myelin bundles containing multiple parallel myelin fibers in the Par after SI (Figures 2I–L; Supplementary Figure 2). The thicker myelin bundles were colocalized with CC1 (a marker for mature OL) expression, suggesting a potential role of more mature OLs in regulating myelin bundle organization (Figure 2N). To identify if SI affected the distribution of mature neurons which may lead to the thicker myelin bundles, we performed immunostaining with antibodies against NeuN (Supplementary Figure 3). The results showed a linear distribution of neuronal cell bodies in Par after SI (Supplementary Figure 3C), which may contribute to more parallel myelinated axons. The cell adhesion protein protocadherin (PCDH) has been reported to regulate the distribution pattern of neurons in cortex (Lv et al., 2022). Specifically, down-regulated PCDH results in a similar linear distribution of neuronal cell bodies (Lv et al., 2022). Consistently, the mRNA level of PCDHB6 was decreased after SI although not to a statistically significant level probably due to a small sample size (Fold change = 0.42; *p* = 0.07; Supplementary Figure 3D). Thus, a reduced PCDHB6 expression may alter the distribution pattern of neurons contributing to the unique pattern of more parallel myelin fibers in the Par after SI.

To further verify SI-induced changes in myelin structure in the Par, we performed diffusion Magnetic Resonance Imaging (dMRI) of SI and control dogs. We quantified the values of fractional anisotropy (FA), mean diffusivity (MD), axial diffusivity (AD), and radial diffusivity (RD), which are commonly used parameters for describing white matter microstructure, in the PFC and Par of SI dogs and group-housed control dogs. The changes from 2 months to 3 months old between the groups were compared because the individual variance. Our results showed a trend of increased FA but decreased AD in the Par white matter of SI dogs (Supplementary Figure 4), indicating more organized myelin fibers, consistent with thicker myelin bundles with more parallel fibers observed by immunostaining (Figures 2D, H, L). No changes were observed for MD and RD in the PFC and Par after SI. The immunohistochemical and imaging results together demonstrate that juvenile SI leads to altered myelin fiber organization in the gray matter of Par but not PFC.

### SI increases the number of mature OLs in Par but not in PFC white matter

**FIGURE 3:**
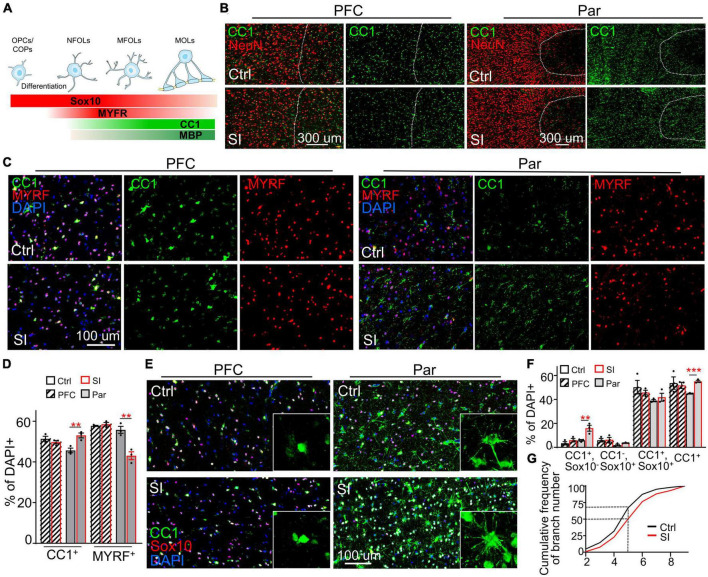
Social isolation (SI) increases the number of mature oligodendrocytes in the white matter of the parietal cortex. **(A)** Schematic representation of the stage-specific markers during OPC differentiation and oligodendrocyte maturation. Markers in bold are analyzed in the present study. **(B)** More CC1^+^ cells in the Par white matter of SI dogs. Co-staining images of CC1 (green) and NeuN (red) in the gray matter (on the left of the interrupted white line) and white matter (on the right of the interrupted white line) of Par and PFC from socially isolated and group-housed dogs. Scale bar, 200 μm. **(C)** Co-staining confocal images of CC1, MYRF, and DAPI in the gray matter of Par and PFC from socially isolated and group-housed dogs. Scale bar, 100 μm. **(D)** Fewer MYRF^+^ labeled immature OLs but more CC1^+^ labeled mature OLs in the white matter of Par after SI. **p < 0.01. **(E)** Triple staining wide-field images of CC1, Sox10, and DAPI in PFC and Par white matter of socially isolated and group-housed dog. Scale bar, 100 μm. **(F)** Statistical results of the percentages of CC1^+^Sox10^+^, CC1^+^Sox10^–^, CC1^–^Sox10^+^, and CC1^+^cells in white matter of PFC and Par. **p < 0.01; ***p < 0.001. **(G)** Cumulative probability plot of branch number of CC1^+^ oligodendrocytes in Par white matter of control and SI dogs.

While myelin-related genes were downregulated in PFC white matter, *ADORA2A*, which inhibits OPC proliferation and promotes OL maturation (Coppi et al., 2021), was upregulated (FC = 31; adj *p* = 0.003) in the Par white matter following SI (Figure 1B; Supplementary Figure 5). Consistently, immunofluorescence staining against NeuN (a marker for mature neurons), the transcription factor CC1, and MYRF [myelin regulatory factor, a marker for premyelinating OL (Huang et al., 2022)] revealed a marked increase in the density and number of CC1^+^ cells in the Par white matter of SI dogs compared with control dogs (Figures 3A–D). In contrast, the number of MYRF^+^ cells in Par white matter decreased after SI compared with that of group-housed dogs (Figure 3C). As a control, there were no obvious differences in the number of CC1^+^ and MYRF^+^ cells in the PFC white matter between SI dogs and control dogs. These results indicate that SI at the juvenile stage induced more mature CC1^+^ OLs together with fewer immature Myrf^+^ OLs in the white matter of the Par but not the PFC.

To quantify the proportion of immature and mature OLs, we performed double immunostaining with antibodies recognizing CC1 and the transcription factor Sox10 (Figures 3E–G), which is expressed throughout the whole lineage including OPCs, with gradually decreasing levels as the OLs mature (Emery et al., 2009). Antibodies specifically recognize OPCs were not available or not working in dog. We quantified the percentages of three populations of OL cells: (i) Sox10^+^CC1^–^ cells, representing OPCs/COPs/NFOLs; (ii) Sox10^+^CC1^+^ cells, representing immature MFOLs; and (iii) Sox10^–^CC1^+^ cells, representing mature MOLs with more cellular protrusions (Figure 3F). Immature OLs (Sox10^+^CC1^–^) showed a characteristic morphology of prominent cell bodies and few filopodia-like protrusions (Figure 3A). Sox10^+^ cells were widely distributed throughout the PFC and Par white matter. OLs in the PFC white matter were relatively more immature than those in the Par white matter based on the protrusion number of CC1^+^ OLs. No obvious changes in the three populations of OL cells were observed in the PFC after SI (Figure 3E). However, the number of CC1^+^ OLs were significantly increased in the Par white matter of SI dogs compared with control dogs (Figure 3F; the percentage of CC1^+^ OLs among DAPI-positive cells of 330∼420 in the Par area of 0.23 mm^2^: 44.8% for control versus 55.1% for SI, *p* = 0.0003). Consistently, the number of cellular processes per CC1^+^ OL was higher in SI dogs than the controls; the cumulative frequency of cellular processes >5 per CC1^+^ OL was 50% in SI versus 30% in control group (Figure 3G). These results show that SI results in more mature OLs specifically in the Par white matter.

### SI results in disrupted blood-brain barrier integrity

**FIGURE 4:**
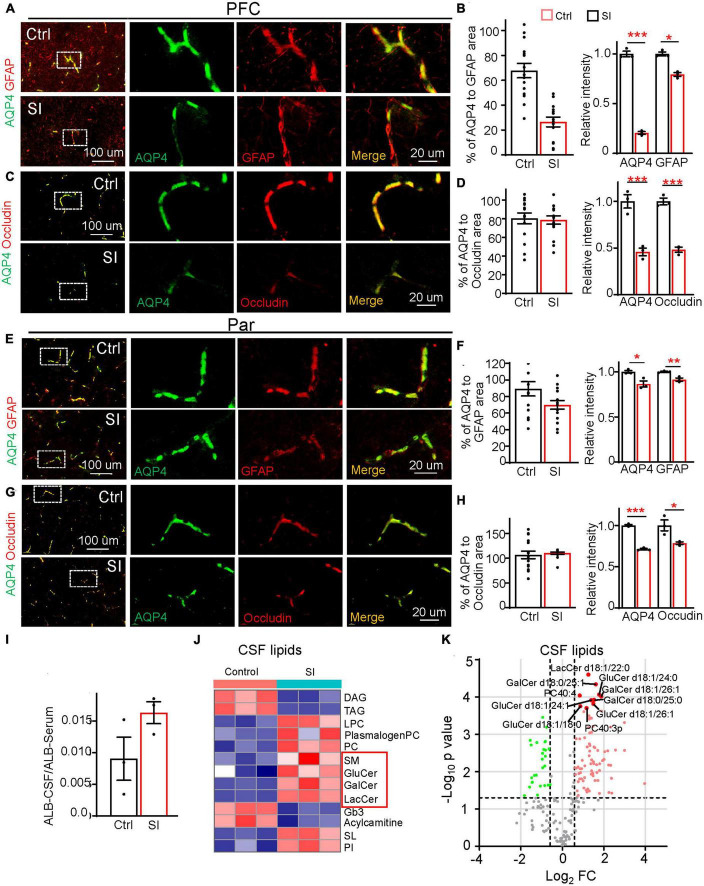
Social isolation of dogs leads to a defective blood–brain barrier and increased lipids in CSF. **(A,C,E,G)** Double staining of AQP4 (green) and GFAP or Occludin in the PFC and Par of socially isolated and group-housed dogs. Scale bar, 20 μm. **(B,D,F,H)** Quantifications of the relative area of AQP4/GFAP, AQP4/Occludin, and the mean intensity of AQP4, GFAP, and Occludin in the control and SI groups (5 GFAP-positive branches per animal). *p < 0.05; **p < 0.01; ***p < 0.001. **(I)** Quantification of the ratio of Albumin level [cerebrospinal fluid (CSF)/Serum] in SI and control groups. **(J)** The heatmap of different lipid class of CSF in group-housed and social isolated dogs. Red box indicates myelin-related lipids which were significantly increased after SI. **(K)** Volcano plots of differentially expressed lipids in the CSF of SI dog brains versus controls by lipidomic analysis.

In addition to myelin-related changes, transcriptomic analysis also revealed a significant decrease in the expression of the gene encoding AQP4 (FC = 0.38; adj *p* = 0.037, Figure 1B), a water channel localized at astrocytic endfeet (a structural component of the BBB), in PFC white matter after SI. To verify whether BBB integrity was compromised by SI, we performed immunostaining and verified a significant decrease in AQP4 protein levels in the PFC and Par (Figures 4A–H). AQP4 was completely colocalized with GFAP around blood vessels in group-housed controls, but it was only partially colocalized with GFAP at the astrocyte endfeet in PFC after SI, suggesting AQP4 is missing at certain areas of GFAP-positive signals (Figures 4A, B). We also examined the expression of other structural components of the BBB, including the basement membrane protein Laminin and tight junction proteins Occludin and Claudin1 (Figures 4A–H; Supplementary Figure 6). All three proteins were expressed and colocalized with AQP4 in blood vessels in the PFC and Par of the control dogs. No obvious changes of expression level were observed for Laminin after SI. However, the expression levels of Occludin and Claudin1 were significantly decreased, similar to that of AQP4, suggesting a disruption of BBB integrity in the PFC after SI (Figures 4C, D; Supplementary Figures 6C, D). In the Par, we noticed that the co-localization of AQP4 with GFAP remained normal (Figures 4E, F); however, the intensity of AQP4, Occludin and Claudin1 at BBB was significantly reduced as in the PFC after SI (Figures 4G, H; Supplementary Figures 6G, H). These results suggest disrupted BBB integrity in different brain regions after SI.

Compromised BBB integrity may lead to the leakage of blood components from blood vessels to CSF. An increased level of albumin in CSF is considered a hallmark of BBB leakage (Algotsson and Winblad, 2007). Consistently, we observed a higher level of albumin, although not reaching significance, in the CSF of SI dogs than in that of control dogs (Figure 4I), possibly due to the greatly varied albumin levels in different individuals and a small sample size. To further examine the effect of disrupted BBB integrity after SI, we performed lipidomic analysis of CSF and found significant increases in membrane polar sphingolipids, including lactosylceramide (LacCer), glucosylceramide (GluCer), galactosylceramide (GalCer), and sphingomyelin (SM), specific components of myelin, in the CSF of SI dogs compared with control dogs (Figures 4J, K). These results indicate that SI results in an increase in myelin-related lipids in CSF, which may be leaked from the affected brain regions through compromised BBB integrity.

## Discussion

In this study, we performed a systematic analysis of multiple brain regions and CSF, from socially isolated and group-housed dogs at the juvenile stage using multiomic and immunochemical analyses. Overall, SI of dogs during juvenile stage lead to a small number of differentially expressed genes in multiple brain regions except the PFC. This could be explained by a few possibilities. First, 3–7 weeks of age of dogs is a critical period for socialization with human beings (Freedman et al., 1961; Scott, 1963). Dog pups separated from the mother at 30 to 40 days during the critical period were more likely to develop a variety of behavioral problems, including fearfulness, noise sensitivity, and excessive barking at later ages (Sargisson, 2014; Dietz et al., 2018). Thus, when dogs experienced SI starting at 2 months of age, their social development was mostly completed. Second, dogs were not completely socially isolated, as caregivers came in twice a day for feeding and cleaning.

Myelination is essential for ensuring efficient connectivity within and among different brain regions; abnormal myelination leads to cognitive dysfunction and abnormal social behaviors (Nave and Werner, 2014; Chen et al., 2020). Prolonged social isolation of adult mice decreases the level of myelin gene transcripts in PFC (Liu et al., 2012). Here, we demonstrated a differential effect of SI on myelin-related processes of different cortices for the first time, i.e., SI during the juvenile stage of dogs induced decreased expression of myelin-related genes in the PFC but more mature OLs in the Par. There are two possible explanations: on one hand, previous studies have demonstrated an important role of neural activity in myelination. Suppressing neural activity during CNS development reduces OPC proliferation (Barres and Raff, 1993) and disrupts the myelination of the optic nerve (Demerens et al., 1996). Conversely, inducing neuronal activity via electrical stimulation or optic genetics promotes OL survival, OL maturation, and axon myelination *in vitro* (Stevens et al., 2002; Ishibashi et al., 2006; Gibson et al., 2014). The PFC is an integrative hub that receives input from all other cortical regions and functions to plan and direct motor, cognitive, affective, and social behaviors (Miller, 1999; Miller and Cohen, 2001; Forbes and Grafman, 2010; Euston et al., 2012). However, during SI, dogs were confined to a cage with much less locomotion, which may lead to decreased neural activities and thus reduced OPC differentiation and OL maturation in the PFC compared with group-housed controls. The Par area is vital for sensory perception and the integration of vision, touch, hearing, and smell (Freedman and Ibos, 2018; Xu, 2018; Medendorp and Heed, 2019). It is possible that the sensory perception would be enhanced to compensate for the broadly inhibited activities of SI dogs. Indeed, dogs showed gradually increased social expectation (heightened attentiveness and increased activities preceding the arrival of caregivers) since the second week of SI (data not shown). As a result, the enhanced sensory perception of hearing and smell in the Par may increase neuronal activity, which promotes OL maturation in the Par.

On the other hand, the effects of SI on OL development in the PFC and Par may differ as the two brain regions develop at different paces, i.e., there are differential myelination progressions in different brain regions of dogs during postnatal development (Hong et al., 2022). Double immunostaining with Sox10 and CC1 revealed that there are more mature OLs in the Par than in the PFC of 3-month-old control dogs. Previous studies have shown that OPCs in white matter form synapses with neuronal axons and establish a microenvironment via excitatory and inhibitory synaptic input from neuronal axons (Karadottir et al., 2008). As OPCs differentiate into premyelinating OLs, they lose synaptic input as well as the expression of glutamate receptors (De Biase et al., 2010). Since the PFC contains more immature OLs and probably more OPCs than the Par, there may be more synapses formed between neuronal axons and OPCs in the PFC. Thus, reduced neuronal activity due to SI may exert a more negative impact on OPC differentiation in the PFC than in the Par. Moreover, transcription and translation occur with different paces at different time points and are detected with different sensitivity, which may explain why reduced myelin gene expression in the PFC following SI didn’t affect oligodendrocyte or myelin fiber number by immunostaining.

In conjunction with more mature OLs, we observed thicker myelin bundles with more parallel myelin fibers in the Par after SI. The reason for this unique myelin fiber pattern which hasn’t been reported before is currently unknown. The more mature OLs and linear distribution of neurons may contribute to the thicker myelin bundles after SI. The linear distribution of neurons may be caused by reduced expression of PCDHB6 in Par; homophilic interaction of PCDHs between neighboring cells from the same progenitor cells have a repulsive effect (Schreiner and Weiner, 2010; Brasch et al., 2019). In addition, vascular endothelin has been reported to regulate the number of myelin sheaths in mouse; increasing endothelin signaling rescues SI-induced myelination defect in the PFC of mice (Swire et al., 2019). It’s possible that SI may increase the expression of endothelin leading to more myelin sheaths in the Par.

Although no obvious disruption of myelin structure in PFC and Par by immunostaining, myelin-related sphingolipids were increased in CSF in the socially isolated group, indicating demyelination, defective myelin formation, or both. Electron microscopy would be needed to ascertain if myelination process in the PFC or Par was affected by SI in future. Clemastine, an antimuscarinic compound, has been shown to enhance OL progenitor differentiation and successfully reverse social avoidance behavior in socially isolated adult mice (Liu et al., 2012, 2016). Given the different effects of SI on OL development in PFC and Par, the effects of clemastine need to be assessed in different cortices. Furthermore, the effect of thicker myelin bundles on neural circuit activity in the Par after SI remains to be clarified.

In addition, we revealed disrupted BBB integrity in both PFC and Par after SI by transcriptomic, immunochemical and lipidomic analyses for the first time. How would SI disrupt BBB integrity? Inflammation and oxidative stress can lead to the overproduction of matrix metalloproteinase-9 (MMP-9) by OPCs, disrupting the integrity of the BBB (Seo et al., 2013). A recent study of SI in juvenile mice shows an increased level of neuroinflammatory cytokine IL-1β and BBB damage in amygdala (Wu et al., 2022). It is not yet clear whether the BBB disruption is due to the overproduction of MMP-9 by OPCs, neuroinflammation, or other unknown mechanisms in the cortices of SI dogs. Further research is needed to fully understand the impact of SI on the integrity of the BBB.

In summary, these findings of shared and distinct changes in multiple brain regions shed new light on the molecular and cellular mechanisms underlying the detrimental effects of SI on the brain. Our study demonstrates the value of dogs as a complementary animal model for a mechanistic study of SI-induced brain development abnormalities and disorders.

## Data availability statement

The datasets presented in this study are deposited in the Genome Sequence Archive (GSA) repository, accession number CRA010898.

## Ethics statement

The animal study was reviewed and approved by the Institutional Animal Care Committee of the Institute of Genetics and Developmental Biology, Chinese Academy of Sciences.

## Author contributions

YQZ conceived and supervised the study. HH, WR, and HZ conducted the dissection. HH conducted the immunostaining. XL, ZZu, and YZ conducted and supervised the MRI data acquisition and analysis. SL and GS performed the lipidomics analysis. CG, LY, ZZh, G-DW, YZ, and YiL conducted and supervised the transcriptomic data analysis. HH and YX conducted the data visualization and wrote the manuscript. JM and YuL provided and maintained the animals. YX, CL, and YQZ revised the manuscript. All authors contributed to the article and approved the submitted version.

## Footnotes

1
http://www.bitplane.com

2
https://pathview.uncc.edu/
