# Supplementary figures and images for: Differential effects of social isolation on oligodendrocyte development in different brain regions: insights from a canine model
Source: Front Cell Neurosci. 2023 Jul 18;17:1201295. doi: 10.3389/fncel.2023.1201295 (PMC10393781; doi:10.3389/fncel.2023.1201295)

Fig. S1

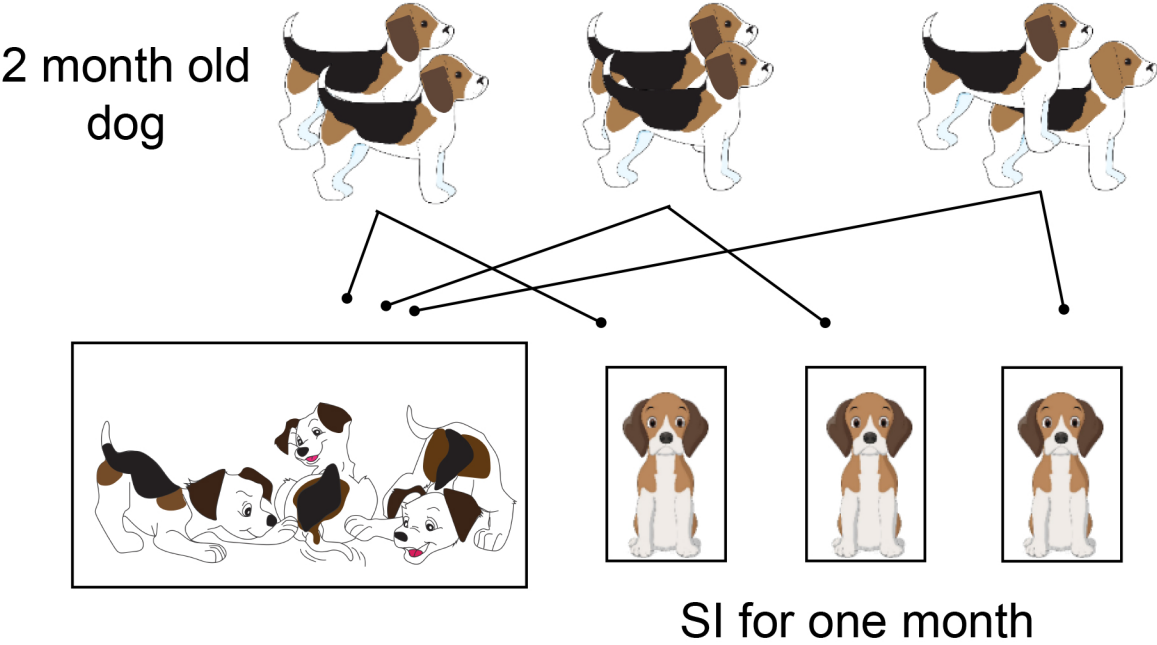

Supplement: Supplementary file 1 [file Data_Sheet_1.PDF]

Fig. S2

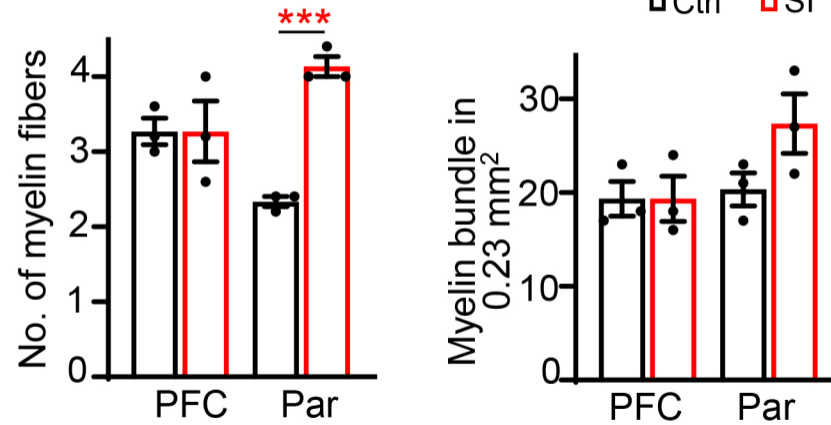

Supplement: Supplementary file 2 [file Data_Sheet_2.PDF]

# Fig. S3

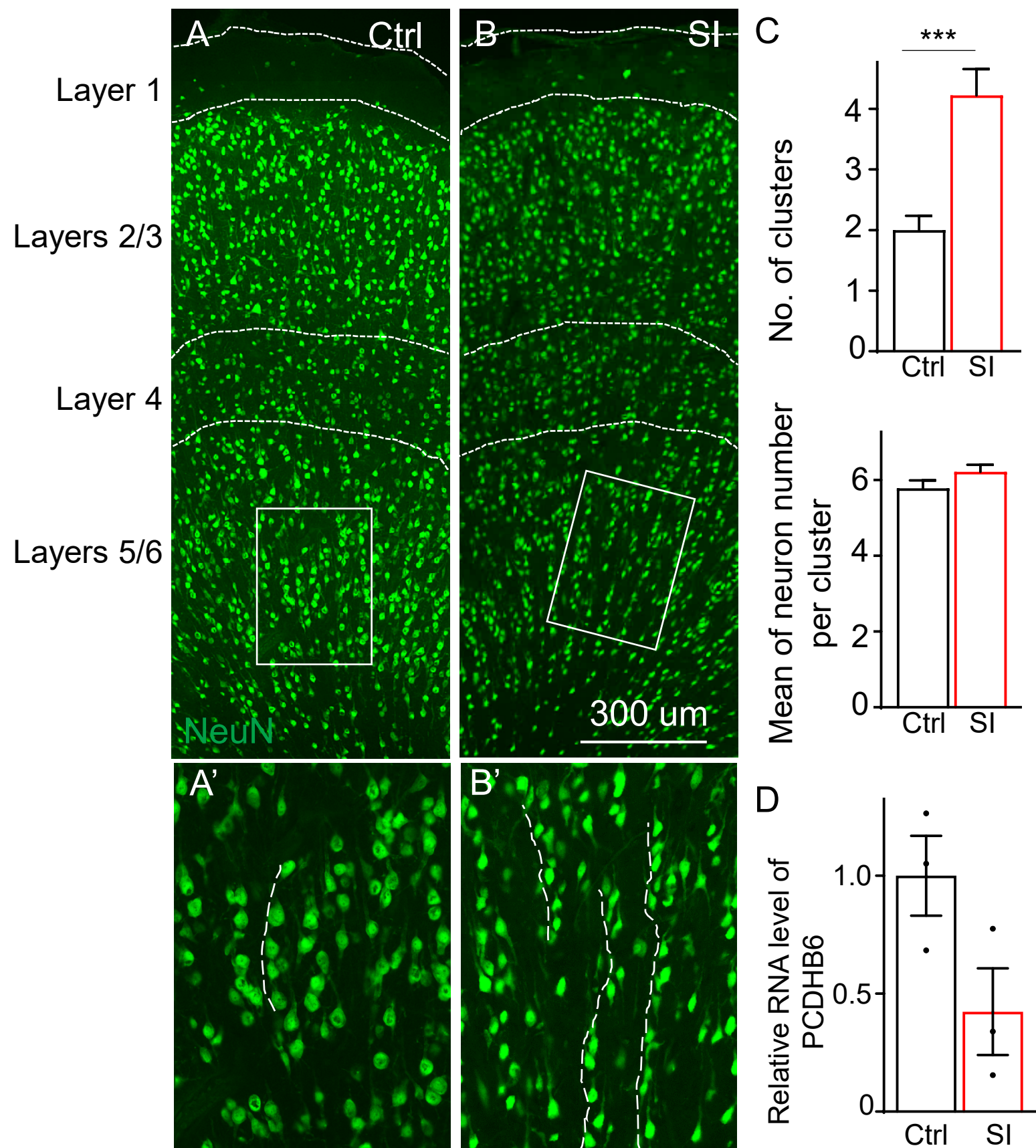

Supplement: Supplementary file 3 [file Data_Sheet_3.PDF]

Fig. S4

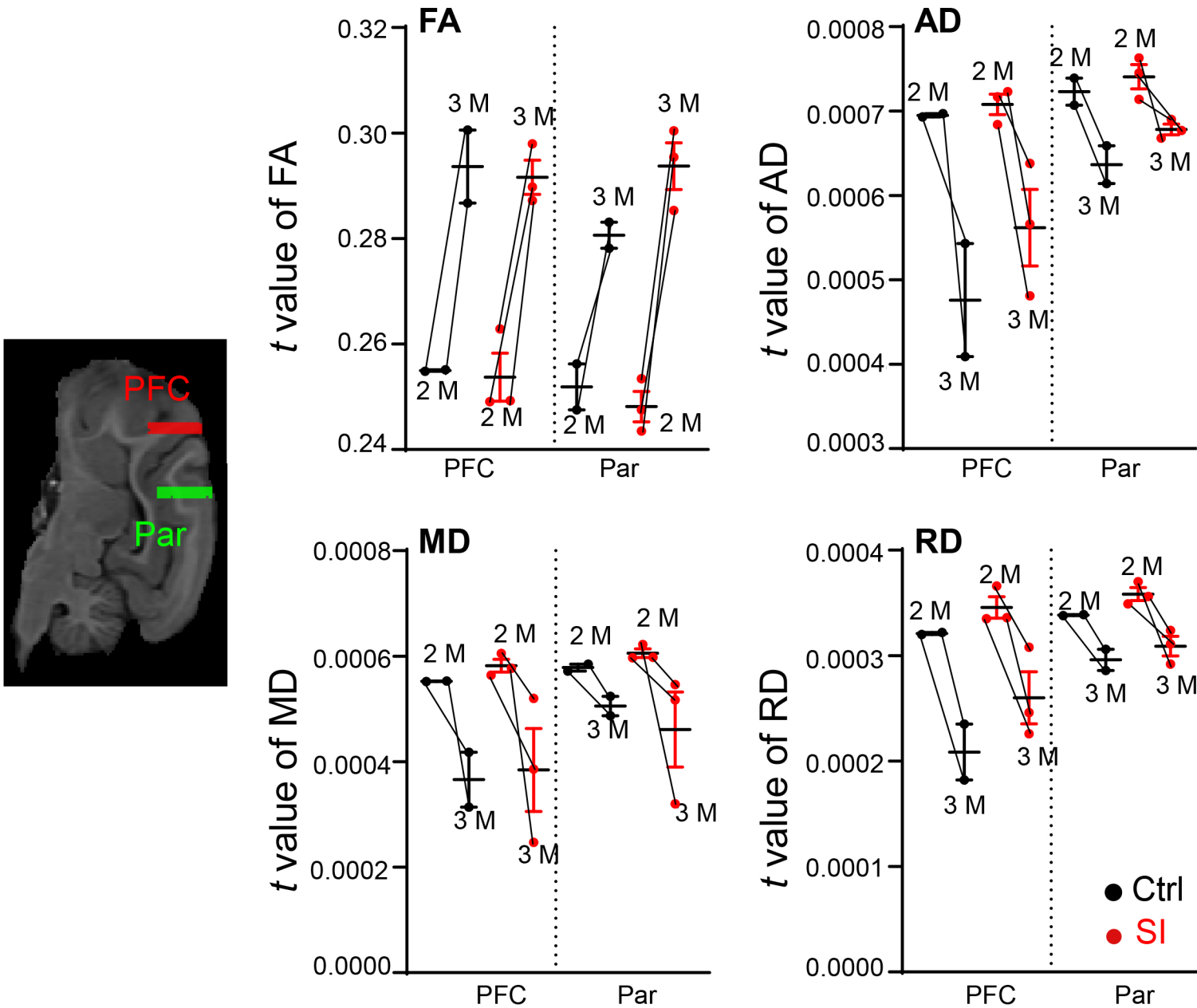

Supplement: Supplementary file 4 [file Data_Sheet_4.PDF]

Fig. S5

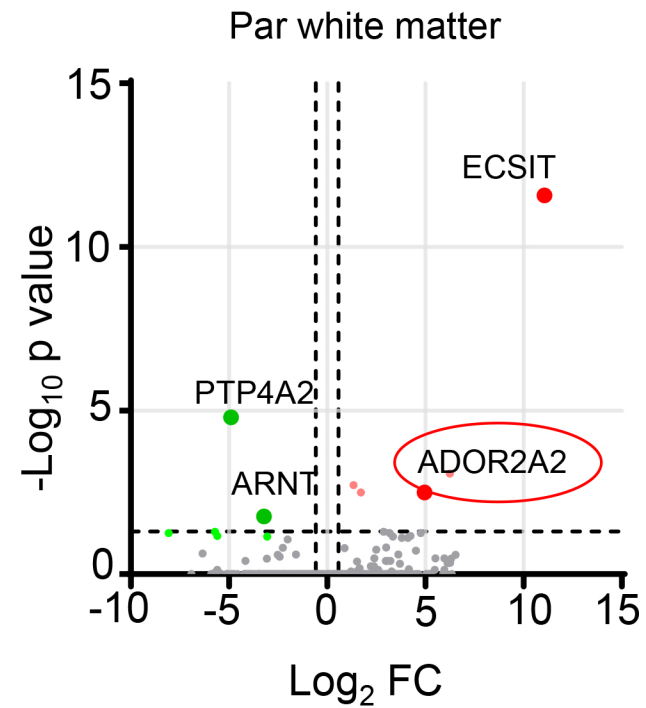

Supplement: Supplementary file 5 [file Data_Sheet_5.PDF]

Fig. S6

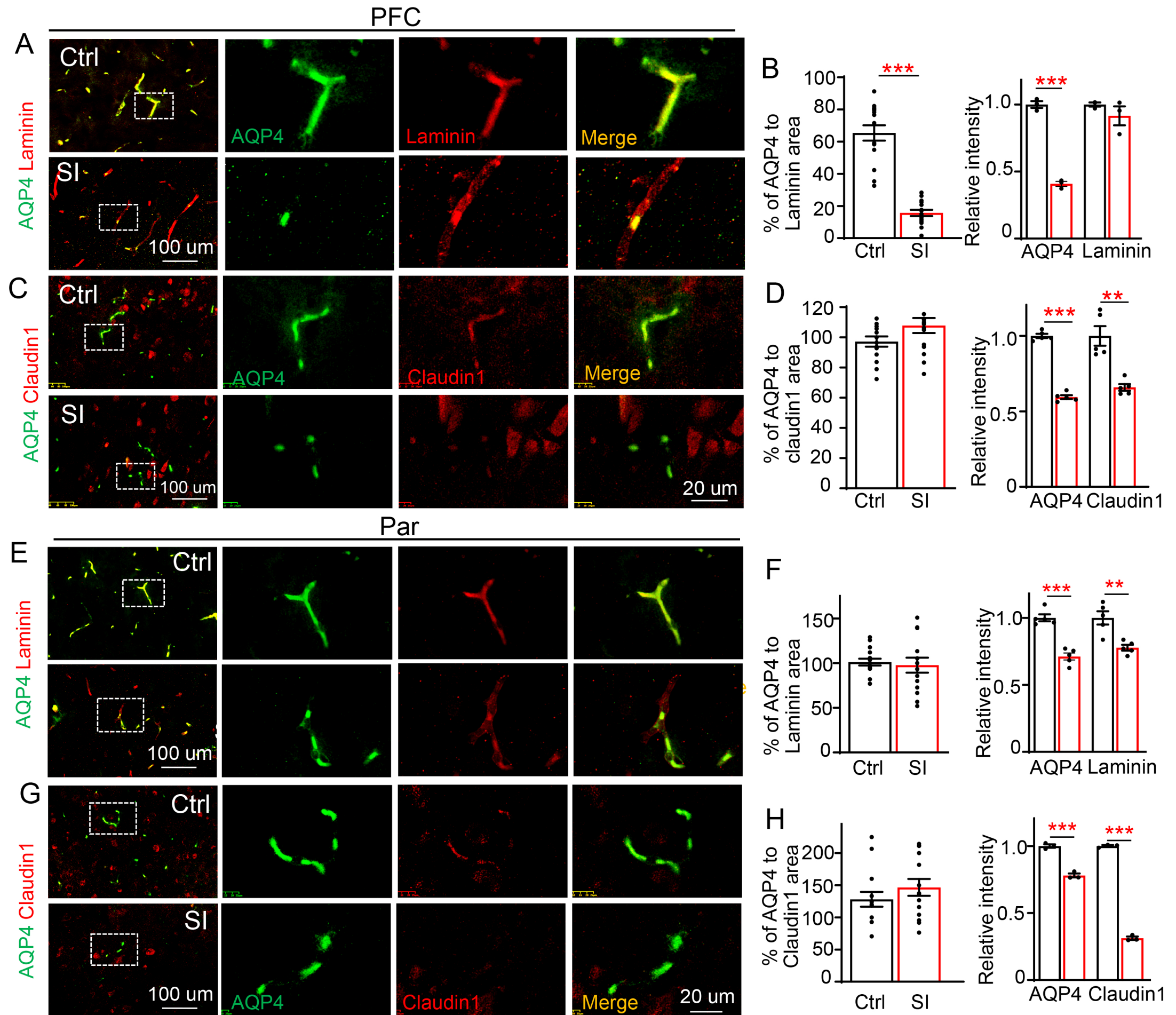

Supplement: Supplementary file 6 [file Data_Sheet_6.PDF]
