# Supplementary material for: Differential effects of social isolation on oligodendrocyte development in different brain regions: insights from a canine model
Source: Front Cell Neurosci. 2023 Jul 18;17:1201295. doi: 10.3389/fncel.2023.1201295 (PMC10393781; doi:10.3389/fncel.2023.1201295)
Supplement: Supplementary file 8 [file Table_2.docx]

***Supplementary Material***

**Differential effects of social isolation on oligodendrocyte development in different brain regions: insights from a canine model**

**Huilin Hong^1^, Chao Guo^2,3^, Xueru Liu^4,5^, Liguang Yang^6^, Wei Ren^1,5^, Hui Zhao^1^, Yuan Li^7^, Zhongyin Zhou^3^, Sin Man Lam ^1^, Jidong Mi^7^, Zhentao Zuo^4,5^, Cirong Liu^8,9^, Guo-Dong Wang^3^, Yan Zhuo^4,5^, Ya-Ping Zhang^3^, Yixue Li^6^, Guanghou Shui^1,5^, Yong Q. Zhang^1,5*^, Ying Xiong^1*^**

^1^State Key Laboratory for Molecular and Developmental Biology, Institute of Genetics and Developmental Biology, Chinese Academy of Sciences, Beijing, China

^2^School of Life Sciences, Division of Life Sciences and Medicine, University of Science and Technology of China, Hefei, China

^3^State Key Laboratory of Genetic Resources and Evolution, Kunming Institute of Zoology, Chinese Academy of Sciences, Kunming, China

^4^State Key Laboratory of Brain and Cognitive Science, Institute of Biophysics, Chinese Academy of Sciences, Beijing, China

^5^College of Life Sciences, University of the Chinese Academy of Sciences, Beijing, China

^6^Bio-Med Big Data Center, Key Laboratory of Computational Biology, CAS-MPG Partner Institute for Computational Biology, Shanghai Institute of Nutrition and Health, Shanghai Institutes for Biological Sciences, University of Chinese Academy of Sciences, Chinese Academy of Sciences, Shanghai, China

^7^Beijing Sinogene Biotechnology Co. Ltd, Beijing, China

^8^Institute of Neuroscience, Center for Excellence in Brain Science and Intelligence Technology, Chinese Academy of Sciences, Shanghai, China.

^9^Shanghai Center for Brain Science and Brain-Inspired Intelligence Technology, Shanghai, China.

***Correspondence:**

Ying Xiong and Yong Q. Zhang

ying.xiong@hubu.edu.cn and yqzhang@genetics.ac.cn

**Keywords: social isolation_1_, dog_2_, myelin_3_, oligodendrocyte_4_, parietal cortex_5_, blood-brain barrier_6_**

**Supplementary Figure legends**

**Supplementary Figure 1 Experimental design of SI studies in juvenile dogs.** Purebred healthy Beagles were obtained from Beijing Marshall Biotechnology Co. Beagle dogs from three different litters were maintained in a natural 12-h light-dark cycle from birth. After weaning at postnatal day 51 (P51), 6 males (weight 2.9 ± 0.3 kg) from three litters (2 from each litter) were reared together in a cage until P60. Then, three males, one from each litter, were housed together in one cage. The other three were housed individually in a quiet location within a building with minimum human activity for one month.

**Supplementary Figure 2 Significantly more myelin fibers in each bundle in the Par but not the PFC.** The number of myelin bundles in 0.23 mm^2^ gray matter area of PFC and Par is presented in the right panel.

**Supplementary Figure 3 Altered arrangement of neurons induced by SI in the Par cortex. (A, B)** NeuN staining (green) was used to label mature neurons in the Par gray matter. A’ and B’ are enlarged NeuN staining images in Layers 5/6 of A and B, respectively, which showed linear arrangement of neurons (indicated by interrupted white lines) after SI. (**C**) Quantification of the number of neuronal clusters as well as the number of neurons per cluster in layers 5/6 of the grey matter per 0.23 mm^2^. The “neuronal cluster” was defined with three characteristics: linear arrangement of neurons; the number of neurons in line ≥ 5; at least one side of the cluster is empty with no presence of neurons. **(D)** Bar plot shows the RNA levels of PCDHB6 in the Par gray matter of control and SI dog brain. Fold change = 0.42, *p* = 0.07.

**Supplementary Figure 4 Statistics of FA, AD, MD, and RD values of ROIs in the PFC and Par between SI and control groups.** The left panel shows the ROIs of the PFC and Par being quantified. The *t* values were calculated by unpaired *t*-test of FA, AD, MD, and RD maps from the two groups.

**Supplementary Figure 5 SI-affected genes and markers of OL lineages in Par white matter.** Volcano plots of differentially expressed genes in the white matter of Par after SI. The cutoff values were set at FC > 1.5 or FC < 0.67 and *p* < 0.05. Signiﬁcantly changed genes are highlighted in red (up-regulated) or green (down-regulated).

**Supplementary Figure 6 Social isolation of juvenile dogs leads to disrupted blood–brain barrier in the Par.** (**A, C, E, G**) Double staining of AQP4 (green) and GFAP, Laminin, Occludin, or Claudin1 in the Par of socially isolated (SI) and group-housed (Ctrl) dogs. Scale bar, 100 μm. (**B, D, F, H**) Quantifications of the relative staining area of AQP4/GFAP, AQP4/laminin, AQP4/occludin, AQP4/Claudin1, and the mean intensity of AQP4, GFAP, Laminin, Occludin, and Claudin1 in the control and SI groups.

**Supplementary Table 2.** **DEGs confirmed by qRT-PCR**

| *Gene* | Full names |
| --- | --- |
| *ECSIT* | evolutionarily conserved signaling intermediate in Toll pathways |
| *ADORA2A* | adenosine receptor A2a |
| *PTP4A2* | protein tyrosine phosphatase 4A2 |
| *ARNT* | aryl hydrocarbon receptor nuclear translocator |
| *MYO5A* | myosin VA |
| *DUSP19* | dual specificity phosphatase 19 |
| *LRRC3B* | leucine rich repeat containing 3B |
| *B3GALT6* | beta-1,3-galactosyltransferase 6 |
| *CNTN2* | contactin 2 |
| *MOBP* | myelin associated oligodendrocyte basic protein |
| *AQP4* | aquaporin 4 |
| *COX7A2L* | cytochrome c oxidase subunit 7A2 like |
| *EGFR* | epidermal growth factor receptor |
| *ErbB3* | erb-b2 receptor tyrosine kinase 3 |
| *NDUFA12* | NADH:ubiquinone oxidoreductase subunit A12 |
| *UQCRH* | ubiquinol-cytochrome c reductase hinge protein |

Note: *ECSIT*, *PTP4A2*, *ADORA2A*, and *ARNT* were tested with the Par white matter. *MYO5A*, *DUSP19*, *LRRC3B*, and *B3GALT6* were tested with the Par gray matter. *CNTN2* was tested with the PFC gray matter. *MOBP*, *AQP4*, *UQCRH*, *NDUFA12*, *COX7A2L*, *ErbB3*, and *EGFR* were tested with the PFC white matter.

**Supplementary Table 3. Primers for qRT-PCR analysis of target genes**

| **Gene** | **Primer sequences (5’-3’)** | **Size (bp)** |
| --- | --- | --- |
| *ECSIT* | TCATCCACTATCCTCGGCAGCAG | 87 |
|  | CGGTCTCCTTGTTGGGCATCAC |  |
| *ADORA2A* | CCCGCGCCTTGGCAGCTCAT | 112 |
|  | GGCCGCCGCTCAGGATGG |  |
| *PTP4A2* | GTGCTCTGCTTCTAAGGAT | 143 |
|  | CATCAGTGTGAGACAAGGT |  |
| *ARNT* | CTTCCTCAGGTAACAGTCAT | 133 |
|  | TGCTCCTCAGTCTCCAAT |  |
| *MYO5A* | GAACCTGATTCTGGAACTGA | 136 |
|  | TGTTAGCAACGACCTTACTT |  |
| *DUSP19* | ACAACATACAGGAGTCAGAA | 150 |
|  | AACGCCAAGACAGAAGTAA |  |
| *LRRC3B* | CCTATGTGGTGTATTATGTGAG | 150 |
|  | TTCTCGGTGTCAGTCTGT |  |
| *B3GALT6* | CAACAGGTTCTCAAGGAATG | 126 |
|  | GTCTATGCCAGCAGTCTC |  |
| *CNTN2* | GTGGTCCTTGTCTCCTCT | 130 |
|  | CCAGTGTCACCTTCTCCT |  |
| *MOBP* | GAATCACATATACCTACACCAG | 104 |
|  | ATACACGCATAAGGCATCA |  |
| *GAPDH* | TGGTGAATGTCGGAGTGA | 137 |
|  | CTGGAACATGTACACCA |  |
| *AQP4* | CTGTCCAGATGTTGAACTCA | 109 |
|  | CTCCACCTGACTCCTGTT |  |
| *COX7A2L* | CAGAAGCACCACCTATCATAT | 128 |
|  | AGGCACACCATCAGACTT |  |
| *EGFR* | GTTATTGCTTCGCCTCAG | 114 |
|  | ATCAGTTCACTTGCTTCTTG |  |
| *ErbB3* | TGCCGACTCTTCAATGAC | 119 |
|  | ACTCCACCATACTGATACTTG |  |
| *NDUFA12* | CCTGAATGGCATCGTTGG | 128 |
|  | GGTACATATTGTTCTGGAGTG |  |
| *UQCRH* | AGGAGGAGGAGGAATTAGTG | 126 |
|  | TCTGTGACCTGGAGGATAC |  |
